# Contribution of Deep-Learning Techniques Toward Fighting COVID-19: A Bibliometric Analysis of Scholarly Production During 2020

**DOI:** 10.1109/ACCESS.2022.3159025

**Published:** 2022-03-11

**Authors:** Janneth Chicaiza, Stephany D. Villota, Paola G. Vinueza-Naranjo, Rubén Rumipamba-Zambrano

**Affiliations:** Departamento de Ciencias de la Computación y ElectrónicaUniversidad Técnica Particular de Loja Loja 110105 Ecuador; Gestión de Investigación, Desarrollo e InnovaciónInstituto Nacional de Investigación en Salud Pública Quito 170136 Ecuador; Facultad de IngenieríaUniversidad Nacional de Chimborazo 27899 Riobamba 060108 Ecuador; Corporación Nacional de Telecomunicaciones—CNT E.P. Quito 170528 Ecuador; Universidad Ecotec, Samborondón 252896 Guayas 092302 Ecuador

**Keywords:** Bibliometric analysis, COVID-19, deep learning, scholarly production

## Abstract

COVID-19 has dramatically affected various aspects of human society with worldwide repercussions. Firstly, a serious public health issue has been generated, resulting in millions of deaths. Also, the global economy, social coexistence, psychological status, mental health, and the human-environment relationship/dynamics have been seriously affected. Indeed, abrupt changes in our daily lives have been enforced, starting with a mandatory quarantine and the application of biosafety measures. Due to the magnitude of these effects, research efforts from different fields were rapidly concentrated around the current pandemic to mitigate its impact. Among these fields, Artificial Intelligence (AI) and Deep Learning (DL) have supported many research papers to help combat COVID-19. The present work addresses a bibliometric analysis of this scholarly production during 2020. Specifically, we analyse quantitative and qualitative indicators that give us insights into the factors that have allowed papers to reach a significant impact on traditional metrics and alternative ones registered in social networks, digital mainstream media, and public policy documents. In this regard, we study the correlations between these different metrics and attributes. Finally, we analyze how the last DL advances have been exploited in the context of the COVID-19 situation.

## I. Introduction

Corona Virus Disease 2019 (COVID-19) is an infectious illness caused by the virus Severe Acute Respiratory Syndrome 2 (SARS-CoV-2). SARS-CoV-2 is the seventh coronavirus known to infect humans, and, like its predecessors SARS-CoV-1 and MERS-CoV, it can cause severe disease. The first human case of COVID-19 was reported in early December 2019 in Wuhan, China [1]. Due to the highly contagious nature of SARS-CoV-2, COVID-19 was designated a Public Health Emergency of International Concern by the World Health Organization (WHO) on January 30, 2020, and officially declared a pandemic on March 11, 2020 [2]. COVID-19 has caused approximately 6 million deaths until 6th March 2022 [3], becoming an unprecedented threat to people’s health and safety due to the extraordinary power of expansion and potential harm.

SARS-CoV-2 attracted the immediate attention of the scientific community. Researchers sought to contribute towards virus detection, spread patterns, and potential solutions to control the global COVID-19 outbreak [4]. According to “*Evidentia Médica*”, a website dedicated to selecting, evaluating, and criticizing the best scientific evidence about COVID-19, at least 118.000 scientific papers have been produced up to March 2021 [5]. Some studies have conducted bibliometric analyses to evaluate the relevance of scientific literature around the COVID-19 pandemic [4], [6]–[14]. Likewise, systematic and meta-analysis reviews have aimed to provide a general vision of the current State-of-The-Art literature related to COVID-19 [8], [15]. These studies present an organized vision of the current literature, explore the main research topics, recognize authors, institutions, and countries currently contributing with COVID-19 related work, and explore connections between research areas.

COVID-19 is being analyzed from different fields to combat the pandemic and its effects on public health and economics. Fields like Statistics, Data Science, Machine Learning (ML), and Artificial Intelligence (AI) can contribute with numerous techniques towards a better understanding of the COVID-19 pandemic [16]. One of the hotspot research areas of AI is Deep Learning (DL). Researchers have used several applications of DL to address issues in different areas related to COVID-19. For instance, DL is used to forecast new cases, transmission analysis with mobility data, or sentiment analysis on social media. This information can help decision-makers to design better strategies and make effective decisions [16]. Furthermore, DL aids diagnosis by employing chest images and cough and breath information from patients. The critical element in these types of analyses is the data, which is known as the “oil” of the new era. Accordingly, the digital economy pursues and values both data accessibility and quality. In this regard, there is a great need for open datasets and studies addressing the classification, consolidation, and identification of gaps in the currently available information [17].

As mentioned before, several bibliometric studies have been conducted to support global research on COVID-19 (see Table 1). However, a lack of a critical pre-processing step could compromise the quality of the presented bibliometric results. An interdisciplinary perspective will allow for a broader and deeper understanding of how each piece of work has contributed to the different research areas. To the best of our knowledge, there is a lack of review work related to the DL-based techniques implemented towards understanding COVID-19. Therefore, the present study aims to:

i) recognize the areas that DL studies have been focusing on, in the COVID-19 context,
ii) identify the main contributions of DL scholarly production against COVID-19,
iii) carry out a bibliometric analysis of the most DL-relevant work applied to the COVID-19 context in terms of traditional quantitative indicators and innovative qualitative metrics that broaden the knowledge about aspects increasing the scientific impact.
iv) share the code and data extracted from several scholarly databases, thus promoting the reuse and extension of future analyses.

**TABLE 1:** Previous Work Carrying Out Bibliographic Analysis of Literature Concerning COVID-19

Ref.	Analysis	Findings and contributions
[4]	The bibliometric analysis gathers information from the Scopus database, including all relevant information on COVID-19 related publications available in the first half of 2020.	The empirical results indicate the domination of health sciences regarding the number of relevant publications, and total citations, while physical sciences, social sciences, and humanities lag significantly. A comprehensive and in-depth approach that consider scientific disciplines in COVID-19 is needed.
[6]	The authors created a knowledge map of the literature regarding SARS-CoV-2. They explored the early status of research on etiology, pathology, epidemiology, treatment, and prevention by using bibliometric methods, citation analysis, and knowledge mapping.	The epidemic situation and data accessibility of different research teams have caused differences in the emphasis of each publication. The authors investigated the area and created the knowledge map of the SARS-CoV-2 literature, and explored the early stages of research on pathology, etiology, epidemiology, prevention, treatment, and control. Also, the authors discussed the remaining knowledge gaps that need to be urgently addressed.
[7]	The authors found 1883 eligible papers in the Scopus database regarding COVID-19. From the corpus, they analyzed publication outputs, countries, institutions, journals, keywords, funding, and citation counts.	The authors recovered a total of 1883 eligible papers regarding COVID-19. Publications came from 94 countries. The most productive countries were China, followed by the USA, UK, Italy, and France. A bibliometric analysis was performed using a machine learning bibliometric methodology.
[9]	The authors used a clustering algorithm to obtain a comprehensive overview of the COVID-19 literature, and to group published articles based on the similarity of their abstracts.	This study showed that an AI-based bibliometric analysis has the potential to rapidly explore numerous academic publications during a public health crisis. The authors identified 19 different topics covered amongst the reviewed publications, with public health and clinical care being the most dominant topics.
[10]	The authors analyzed the global literature about COVID-19 published between 2019 and 2020 and indexed by the Web of Science (WoS) collection database by using bibliometric analysis and the VOSviewer tool.	The study gathered traditional bibliometric indicators of about 3,626 publications regarding COVID-19. The main indicators analyzed are: mean citation count of the top 100 most cited, highest-ranking journal, and most-cited journal among others.
[11]	The authors searched the COVID-19-related literature officially published and included in the WoS database, and the work submitted to four preprint platforms: bioRxiv, medRxiv, Preprints, and SSRN.	This study comprehensively analyzes the COVID-19-related literature based on the WoS database, bioRxiv, medRxiv, Preprints, and SSRN. Many COVID-19-related reports were produced by researchers from the USA and China. The authors analyzed global trends in COVID-19 research, including institution distribution and research hotspots.
[12]	The authors used a metadata provided by the Scopus database to analyze the type of publication, collaboration index, the most productive countries, scientific journals, the institutions with more publications on the subject, and indicators of citations and impact.	A total of 547 published documents were identified, and important characteristics were analyzed. The journals with the most published documents were: The Lancet, British Medical Journal, The Journal of Medical Virology, and Wuhan University. The latter was identified as the institution with the highest leadership regarding the number of publications.
[13]	The authors compared research between English and Chinese studies by doing a bibliometric analysis considering publication metadata such as titles, authors, co-cited authors, journal source, keywords, affiliations of authors.	English and Chinese language publications enabled doctors to exchange information with others. Also, they provided complimentary educational approaches for local doctors in order to understand the essential information to manage COVID-19. Network maps were generated to evaluate the collaborations between different authors, countries/provinces, and institutions.
[14]	The data analysis was carried out with SciMAT software. The information was obtained from WoS Core Collection, and Scopus databases. The data was gathered from December 1, 2019, to September 6, 2020.	This study used bibliometric analysis to evaluate the publications on COVID-19 linked to environmental studies. The analysis was based on a collection of 440 articles broken down into six main themes: (1) reduction in air pollution and level of water pollution; (2) the relationship of wind speed, ultraviolet radiation and humidity with the rate of infections; (3) the effect of the pandemic on the food supply chain and waste habits; (4) wastewater monitoring; (5) AI and smart devices usage in citizen mobilization monitoring; and (6) the lessons gleaned from the pandemic that help define actions to mitigate climate change.
[16]	This study analyses forecasting models available in the literature. The authors studied various statistical, analytical, mathematical and medical parameters such as the COVID-19 impact on environmental factors, incubation period, the impact of quarantine and others.	This study categorizes forecasting models into four major sets which include big datasets accessed from WHO/National data sources, social media/other communication media data, stochastic theory/ mathematical models and data science/Machine learning techniques.
[17]	The survey categorizes COVID-19 open-source datasets based on data type and dataset application. Medical images, textual data, and speech data formed the main data types.	The authors compared research work of COVID-19 open-source datasets. The applications of open-source dataset included diagnosis, infection estimation, mobility and demographic correlations, non-pharmaceutical interventions (NPI) analysis, and sentiment analysis.

In summary, this study contributes to a better understanding of DL techniques and their latest advances towards fighting public health issues. Moreover, the bibliometric analysis can help to understand the dynamics around scientific impact, how sensible topics for humanity increase the interest of the research community, and move resources to counteract adverse effects.

The rest of the paper is organized as follows: Section II presents the background. In Section III, we analyze the related work on Bibliometric and AI Techniques. In Section IV, we describe how the process was conducted. Section V discusses the quantitative and qualitative results. Finally, Section VI draws up the main conclusions.

## II. Background

This Section presents the main concepts in the context of our research to aid in the understanding of each section. First, we describe the types of research oriented to scientific production analysis, and then we introduce the DL technique and related terminology in the current COVID-19 situation.

### A. Types of Literature Analysis

1) *Bibliometric Analysis* is defined as applying mathematical and statistical methods to analyze the distribution patterns of scientific production within a topic, research area, institution, country, and other disciplines. Bibliometrics has been used in different fields to determine quantitative, qualitative, and structural aspects of scientific publications [18]. Such outcomes are handy for promoting researchers, elaborating proposals, funding applications, and identifying hot-spots, trends, references for cooperation, amongst others.
2) *Literature survey* is a systematic method for gathering information to construct quantitative descriptors about the research subject. Once the information is gathered, data is organized and grouped into evidence to develop the claims. This can be done by building the groups chronologically, thematically, or in combination. Following that, researchers compose the reasoning patterns and maps to create simple arguments. Finally, a discovery argument is built for what is known about the subject of research [19].
3) *Literature review* is a traditional method of summarizing current and historical research topics to organize the published literature and qualitatively identify future trends, gaps, and relationships between concepts. However, this type of research suffers from drawbacks such as lack of transparency in research methods and selection criteria. This type of review discusses concepts and general ideas instead of analyzing data or findings. Moreover, literature reviews are regarded as subjective studies prone to show bias [20].
4) *Systematic and meta-analysis reviews* are situated at the top of what is known as the “Evidence Pyramid” [21]. A systematic review is referred to as the entire process of collecting, appraising, and synthesizing evidence aiming to answer a well-structured question with a rigorous and transparent research protocol [20]. Meanwhile, a meta-analysis is a statistical method used to combine numerical results of independent studies derived from the systematic review. Hence, a systematic review can include a meta-analysis, but a meta-analysis is always part of a systematic review. If a quantitative approach is carried out, both types of reviews are generally conducted.

### B. Deep Learning and Related Terminology

1) *Artificial Intelligence* (AI) is the discipline of Computer Science that focuses on expanding human limits. Nowadays, AI has transitioned from theory to several AI-powered tools thanks to the rapid technological advancement and exponential increases in large datasets [22].
  In addition to analyzing large volumes of data, AI methods are capable of dealing with complex problems by providing suitable solutions for clinical practice [23]. Likewise, some AI methods have the potential to exploit meaningful relationships within a dataset, which can be used in several scenarios, especially in many biomedical health tasks [24], e.g., brain tumor detection, cancer detection, among others [25]. Furthermore, several AI-based algorithms have been approved in the last decade by the Food and Drug Administration (FDA) of the United States and could therefore be implemented [23].
  Motivated by the worldwide COVID-19 crisis, AI is being used to find solutions quickly. AI-based techniques for automatic medical detection and diagnosis, have proved successful for COVID-19 cases [26]. In only a few weeks, a number of researchers came up with several mathematical models to predict the transmission [27], and other researchers have used radiology images for COVID-19 detection [26]. Consequently, AI techniques could be positioned in clinical administrations to help fight COVID-19. In this regard, among the most popular applications of AI are forecasting and image-based diagnosis, thanks to AI methods that provide flexibility and domain adaptation at a low cost [27].
2) *Machine Learning* (ML) is an innovative approach with broad applications for prediction. ML is a subfield of AI, in which machines analyze data related to a specific task and learn from that data to build a model. In this way, when new data is provided, the system can automatically identify patterns and make inferences [28] by predicting or grouping an unknown observation. Usually, ML methods provide accurate features instead of a traditional approach based on explicit computation.
  ML methods are increasingly becoming more reliable and widespread in the biomedical domain. As new data emerges, ML advances to benefit clinical decision-making and computer-aided systems [29]. In particular, the study of medical images has experienced significant progress because ML systems can automatically extract the necessary characteristics to make a correct diagnosis [30]. Last year, ML techniques were applied to the COVID-19 pandemic to identify high-risk patients and their mortality rates. These risk factors can also be analyzed according to age, social habits, location, and climate. Furthermore, ML is used to understand the nature of SARS-CoV-2 and predict the problems in the pandemic. Similarly, ML techniques have been used in pharmaceutical areas to predict potential outcomes of existing drugs towards the treatment of COVID-19.
3) *Deep Learning* (DL) has become a crucial breakthrough of AI [31]. DL includes a subset of ML methods, those inspired by the structure and function of the human brain, i.e., Artificial Neural Networks (ANNs). DL models are categorized into non-pre-trained and pre-trained [32]. Non-pre-trained models are trained from scratch, so they need massive datasets and are prone to over-fitting. In contrast, pre-trained DL models are already trained with large datasets. DL focuses on the mining, analysis, and recognition of patterns from data [29]. Unlike other ML methods, DL ones allow for automatic extraction of semantic features [30]. Due to this ability, the performance of pre-trained models is higher in most domains than the traditional methods [32].
  DL techniques deliver an increased performance and reveal image features that are not apparent in the original images [25]. Hence, DL has been increasingly used to segment and classify biomedical images [33]. Furthermore, DL can lead the clinical decision-making and automation of preliminary diagnoses, which is of tremendous significance in the medical community [34]. Specifically, Convolutional Neural Networks (CNN) are used to enhance image quality in low-light images. CNN are also applied for diagnosis and prognosis via images and automatic labeling of images from videos [25].
  Since 2020, DL methods have effectively distinguished COVID-19 images from images of healthy patients [25] and other types of pneumonia. Studies based on DL have been helpful for COVID-19 diagnosis and prognosis [35]. According to Tabik *et al.*, an increasing number of recent research claims to achieve impressive sensitivities (> 95%), far higher than expert radiologists [36].
  Nonetheless, despite the promising results of DL, there are some limitations or problems. For instance, diagnosing and screening COVID-19 could require large-scale labeled imaging data. To combat this, Peng *et al.* integrated medical imaging and natural-language processing to annotate large-scale medical images required by DL models [35]. This additional data can improve the performance of a DL model to classify COVID-19 versus non-COVID-19 lung disease. Another disadvantage of DL methods are the lack of transparency and interpretability, i.e., it is difficult to determine what imaging features are being used to determine the output. In Ref. [37], the authors used a heatmap to visualize the critical regions. However, heatmaps are still insufficient to identify the model’s features that distinguish between COVID-19 and other lung diseases.

## III. Related Work

Researchers from various disciplines such as biology, medicine, computer science, socio-economics, and tourism are resorting to bibliometric analysis to mitigate the adverse effects of COVID-19 [4], [6], [8], [10]–[14]. For instance, Shuja *et al.* contribute by surveying and classifying open-source datasets related to the COVID-19 pandemic and highlighting the data that should be publicly available or extended, especially related to CT-scan and X-ray images for higher accuracy of DL techniques [17]. These extensive studies use innovative bibliometric approaches such as VOSviewer/SciMAT network analysis, Venn diagrams, and binary logistic regression-based text mining. Table 1 presents more details about contributions of bibliometric-analysis-related work.

Regarding computer science contributions, and specifically, regarding AI techniques application to COVID-19 mitigation, there have been comprehensive research studies [15], [38], [39] focused on different COVID-19 aspects such as prediction, diagnosis, image recognition, among others. To this end, the accessibility of information plays a crucial role in the realization of descriptive, predictive, diagnosis, and prescriptive analytics. Indeed, the combination of AI techniques and rich datasets provide a powerful tool to assist human decisions. Moreover, some studies, such as [38], give a clear and concise analysis of intelligence techniques and methods to combat various pandemics based on medical information. These investigated techniques present advances in analyzing medical data with reasonable accuracy. More details about relevant AI-related work in the context of COVID-19 are presented in Table 2.

**TABLE 2:** Previous Work Doing Literature Analysis Regarding the Application of AI Techniques in the COVID-19 Context

Ref.	Analysis	Findings and contributions
[15]	The authors performed a meta-analysis of the current state-of-the-art regarding AI and COVID-19 by using various print and pre-print publication repositories.	This study helps the researcher to understand the broad spectrum of AI to combat COVID-19. The authors highlight four primary areas of AI applications: (1) diagnosis and prediction, (2) epidemiology, (3) molecular study, drug design and treatment, and (4) commerce, business, governance, education, and training.
[38]	The authors survey state-of-the-art work regarding the application of intelligent systems such as ML and DL for COVID-19 prognosis. They addressed a systematic review process to analyze 133 papers.	ML and DL have been mainly used to detect, predict, identify, and screen COVID-19. Specialized methods and algorithms of ML and DL still need to be applied with COVID-19 data for better performance analyses. The authors highlight that one of reasons is the lack of data. So, more real-world datasets with updated COVID-19 data need to be created.
[39]	The authors survey the current state of DL’s contribution to control the COVID-19 pandemic and the critical limitations of DL for COVID-19 applications across Natural Language Processing, Computer Vision, Life Sciences, and Epidemiology domains.	This study covered some of DL’s most pressing limitations, including interpretability challenges, generalization metrics, learning from limited labeled datasets, and data privacy. For instance, the authors highlighted that DL systems must be designed with Human-AI interaction in mind. Moreover, the authors found that most DL research in COVID-19 focuses on novel combinations of learning tasks or on building new datasets and annotation protocols.

The related work analyzed here have been published in 2020, as described in the following Section IV. However, the investigation into AI applications to combat COVID-19 is still ongoing, like the recent publication [40] where a study about COVID-19 risk estimation for educational institutes is presented.

As described, previous studies have focused on generic bibliometric analysis across the large spectrum of COVID-19 literature. Computer Science has traditionally contributed to several sectors, including medicine. In this regard, researchers of this discipline will need to understand the contributions of DL towards the COVID-19 fight to continue working on this ongoing research, learn lessons to future applications and know about the leading bibliometric indicators of this specialized scientific production. We aim to cover this gap by analyzing the scientific production around DL-based techniques implemented towards understanding COVID-19.

## IV. Methodology

**FIGURE 1.**
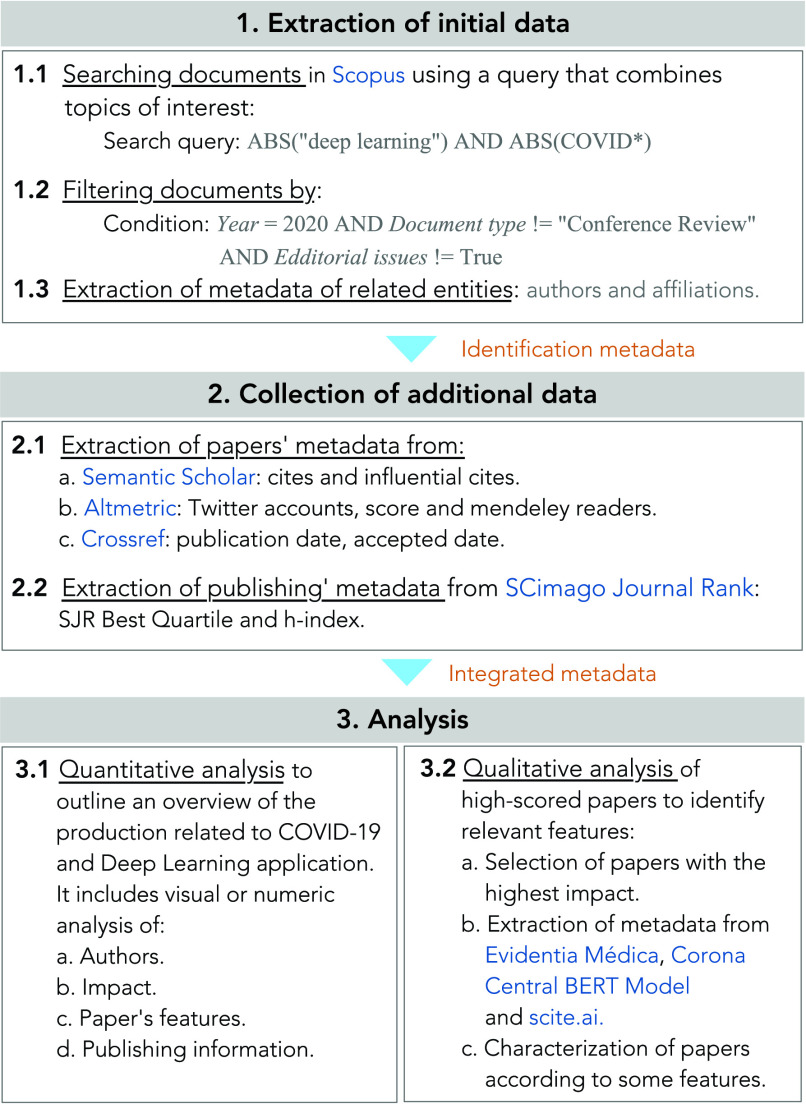
Three-stage process followed for search strategy, extraction, collection, and analysis of data.

We design a three-stage process to identify and analyze the literature related to the terms DL and COVID-19. First, we execute an initial search in the Scopus database to extract the bibliometric information about documents of our interest. Later on, we retrieve data automatically from Semantic Scholar, Altmetric, Crossref, and Scimago Journal Ranking (SJR) to find additional metadata of the papers found in Scopus. Finally, from the bibliometric data retrieved, we carry out two types of analysis: i) quantitative, guided to get a general perspective of the scientific production in the scope of our study, and ii) qualitative, oriented to determine the contribution and exciting features of the most cited or popular documents. Fig. 1 summarizes the workflow of the three-stage process and the main tasks carried out in each one.

### A. Extraction of Initial Data

We carry out three steps to gather the bibliometric data of the published work related to DL and COVID-19:

1) We identify the base literature by using the Scopus Search API and the search string <ABS(“deep learning”) AND ABS(COVID*)>. This search was made in the abstract of the documents ( $ABS $) to retrieve documents closely related to the domain of interest. As a result, we obtain 480 documents.
2) We select the documents by applying three filters: $publication year = 2020$, $document type \neq ``Conference Review ''$ and $Editorial Issues \neq True$. The first filter is established to analyze the scientific production of an entire year, i.e., 2020-year. The second filter is used to exclude documents that are not specific research types. Finally, the third filter was applied to discard documents with editorial issues like *retracted* articles. After applying these filters, we obtain 316 documents.
3) We use the Affiliation and Author Retrieval APIs to obtain additional information about authors and their corresponding affiliations of the selected papers. As a result, we acquire 1,654 unique authors and 710 different affiliations. Metadata obtained from each author include *h-index*, knowledge areas, and the number of years publishing in Scopus (i.e., career length). For each affiliation, the extracted information includes the name of the institution and its registered home-institutional country.

### B. Collection of Additional Data

We implement automated methods to acquire additional information about each paper and its respective publisher. To extract additional metadata of each paper, we use the APIs provided by three different sources: Semantic Scholar, Altmetric, and CrossRef. The code and data extracted from each complementary source is available in the COVID19-DL Github repository.^1^

• *Semantic Scholar* is a project powered by the Allen Institute for Artificial Intelligence, which provides open access to scientific research. This project indexes journals included in the Directory of Open Access Journals (DOAJ). Hence, the number of citations recorded by this project for a paper could reflect most of the publications citing such work. Furthermore, this project provides the “influential citations” score [41], which identifies citations where the cited work has a significant impact on the citing publication. Influential citations are determined by utilizing an ML model and analyzing several factors, including the number of citations one publication receives and the surrounding context for each of them.
• *Altmetric* provides programmatic access to the metrics data associated with articles [42]. Altmetric returns a record of the online attention for an individual piece of scholarly content. By using the Altmetric API, we obtain information such as (1) the number of Twitter accounts that have tweeted this publication; (2) total reader counts that represent the total number of unique users who have saved this article in Mendeley, CiteULike, or Connotea; and (3) the Altmetric Attention Score (AAS) for each paper that measures the online presence of published articles [43].
• *CrossRef* provides detailed information about the evolution of a paper, i.e., critical dates as document creation, acceptance, publication, and indexation. In this work, we refer to each paper’s creation date to determine how many months a paper has been available for public access and citing.

^1^https://github.com/jachicaiza/COVID19-DL

The additional bibliometric data regarding citation and impact obtained from Semantic Scholar and Altmetric help us build a complementary and alternative perspective to the information gathered from Scopus.

To extract further metadata of the publishers and publishing sources, we use the 2019 ranking of journals and proceedings provided by SCimago Journal Rank (SJR). We use this information as it was the only available at march 5th 2021 (the cut-off date of data collection). The SJR metadata includes information on the SJR Best Quartile, *h-index*, and country of each source.

### C. Analysis

Once data from different sources (or databases) is consolidated by following the ETL (Extract, Transform, and Load) process, data analysis is carried out by splitting it into quantitative and qualitative analyses.

#### 1) Quantitative Analysis

This analysis aims to study the relevance of research documents based on numerical indicators. In our study, we want to investigate research performance in the context of DL and COVID-19. Traditionally, bibliometrics regarding research impact involves ranking the research documents based on the occupied quartile, author’s *h-index*, or the number of citations.

Upon further analyses into these indicators and others, we aim to find answers to the following queries:

• *Is there a relation between the publication impact (i.e., number of citations and h-index) and the author’s career length?*
• *What country affiliation stands out in the research production about DL and COVID-19?*
• *How has the traditional citation count metric evolved for papers applying DL techniques in COVID-19 fighting?*
• *What impact do indicators obtained from social media or bibliographic platforms have over citation counts?*
• *Which type of document is preferred to present results?*
• *Which access mode (open access or based on subscription) is dominant?*
• *What topics and trends are the most common in the investigation against COVID-19?*
• *Which publishers and sources concentrate most of the research production in the scope of our study?*
• *What are the authors’ preferred publishing sources (journal or conference and proceedings) to submit their work?*
• *How many citations have papers in the context of our study received per publisher?*
• *Where are the publishing sources’ headquarters? What quartile ranking do they have?*

Proper consolidation of the information from different sources and suitable queries enable analyses to answer these and other questions. In our case study, metadata has been consolidated in a MySQL database and exported in CSV formats to be analyzed and visualized in different tools like Tableau, VOSviewer, R and Python.

Regarding statistical analyses carried out in R [44], data is tested for normality of the distribution by Shapiro-Wilk’s test with a confidence interval of 95%. Citation count, distribution date, and creation date are not normally distributed. Statistical comparisons are performed by Mann–Whitney U test and Spearman’s rank correlation coefficient.

#### 2) Qualitative Analysis

The quality of the published research is analyzed regarding the impact it generates on different sectors. In work concerning COVID-19, such sectors include the research community, public health, governments, and, in general, society. In contrast with quantitative analysis, qualitative evaluation usually addresses descriptive reports, which mainly answer questions regarding research methods, orientation, or the contribution a paper generates. In our study, this analysis aims to identify the critical characteristics of a published work that have led it to achieve a high citation impact and social network attention.

We identify a set of papers that have achieved leading citation impact by choosing the top-10 papers with the highest citation scores in Scopus and Semantic Scholar until February 2021. Furthermore, we take the top-10 papers with eminent attention on social networks as reported in the Altmetric database. By joining these three sets, we obtain 22 unique papers.

Additionally, it was also imperative to consider the papers that, due to the short time elapsed since their publication date until the date of this analysis, have not achieved a high citation score to appear in the top-10 list. To address this unfair situation, we merge the set of 22 papers with the papers having fulfilled the following criteria *“months since their publication <= 3 and (Scopus_citation_count >= 5 or SematicScholar_citation_count >= 5)”*. In the end, we obtain a list of 27 papers which we will refer hereinafter to as the top-27 papers.

From the list of top-27 papers, we analyze the following features: contribution area, scope, medical application, influence, citation context, multidisciplinarity, and technical information, such as prediction task, DL method and model, and performance reported by each paper.

##### a: Contribution Area

“*Evidentia Médica*” [5] and *CoronaCentral BERT Model* [45] provide the topic type classification based on six and thirty-one categories, respectively, as described below:

• *Evidentia Médica*: Diagnostic, Treatment, Etiology, Prognostic, Epidemiologic Models, and Guidelines.
• *CoronaCentral*: Clinical Reports, Communication, Contact Tracing, Diagnostics, Drug Targets, Education, Effect on Medical Specialties, Forecasting & Modelling, Health Policy, Imaging, Healthcare Workers, Immunology, Inequality, Long Haul, Infection Reports, Medical Devices, Misinformation, Model Systems & Tool, Molecular Biology, Non-human, Non-medical, Pediatrics, Prevalence, Prevention, Psychology, Recommendations, Risk Factors, Surveillance, Therapeutics, Transmission, and Vaccines.

##### b: Scope

From each paper, we manually obtain the geographic location where the research was conducted or to which it can be applied.

##### c: Medical Application or Usability

We manually identify the capability of a paper to immediately contribute towards a medical application by analyzing the abstract, results, and discussion of each of the top-27 papers. Research that needed further work to be applied in a medical field was classified as not valid for medical purposes.

##### d: Influence

In addition to the traditional citation count metric, it is interesting to consider all information generated on the web around one research document, which can be read, visualized, linked, shared, downloaded, reviewed, mentioned, commented, followed, and disseminated. This multiple possibilities can give us valuable insights into the research impact and influence on society, governments, academia, public and private organizations. For instance, *Author Level Metrics* [46] are broader metrics on citations and the digital footprint that scientific documents left on the Internet. By leveraging the *Altmetric.com* API, we aim to catch part of this digital footprint from social and digital communication media or public health policies issued by authorities and available on the Internet.

##### e: Citation Context

The citation count influences the well-known *h-index*. Nevertheless, it is interesting to know the context in which a document is referenced. Generally speaking, one citation can be positive or negative. The latter can occur with controversial papers that raises the research community’s attention and receive negative criticism. The traditional citation count metric cannot catch this effect and may unfairly increase the author’s *h-index*. We manually obtain the citation context by using the *scite.ai* website [47]. This website applies AI techniques to address this goal based on: *Supporting*, *Mentioning*, and *Contrasting* categories. Additionally, *scite.ai* provides the specific section whereby the research document is referenced, the type of publication (article, book/book chapter, pre-print, other types), and discriminates between independent and self-cite references.

##### f: Multidisciplinarity

We determine the composition of each research team regarding the areas in which they have published during their career. We use the Scopus database to obtain the subject area categories from every publication. We group these subject areas into four knowledge areas, i.e., Life Sciences & Biomedicine, Technology, Physical Sciences, and Social Sciences. We determine the percentage of authors that contribute to the publication of an article in each subject area. We further determined the number of published papers per author and per subject area.

##### g: Prediction Task

In ML, there are three automatic tasks: *regression*, *classification*, and *clustering*; each task has a specific goal and application. *Regression* aims to predict a numeric value for an event or observation, e.g., forecasting the number of COVID-19 deaths in a period. *Classification* seeks to label an instance or data according to a set of previously established categories. In biomedicine, an application of the classification task of digital images is to detect different medical conditions, such as pneumonia and tumors. Finally, *clustering* is similar to classification, but instances are grouped in unknown categories.

##### h: DL Method and Model

There are three methods of DL that are the most popular: convolutional neural networks (CNN), recurrent neural networks (RNN) and feed-forward neural networks (FNN). Each method or family of algorithms can be used to automate tasks such as classification and regression. Likewise, several models are pre-trained to speed up pattern recognition on data by leveraging specific architecture based on a DL method. Pre-trained models are the basis for tuning or extending an architecture to do some tasks more accurately. For example, to classify images, there are some pre-trained models such as *ResNet* or *Inception*. In this study, we identify the method and models proposed or reused in each selected paper.

##### i: Performance

We analyze in detail the content of each work to distinguish the relevant components of the performance of each paper by identifying the following characteristics: (1) Architecture, the name of the designed model or architecture evaluated by the authors, which presented the best performance; (2) Data type, define the nature of the data; (3) Data size, amount of data used for experimentation and validation, expressed in thousands; (4) Number of classes, identify the number of classes or categories authors used to group their data with a DL approach (e.g., two classes of CRX images: healthy or COVID-19); and (5) Evaluation metrics, known metrics used to evaluate models with an ML approach, namely, accuracy, specificity, positive predictive value, sensitivity, F1 score, area under the curve (AUC), among others.

## V. Results and Discussion

### A. Quantitative Analysis

In this subsection, we present the results about quantitative analysis grouped into four main categories: (1) *Authors*, (2) *Impact*, (3) *Paper features*, and (4) *Publishing information*. The visualization project is available in the Public Tableau site.^2^^2^https://archive.org/services/purl/dl4covid/viz

#### 1) Authors

##### a: Author’s Attributes and Paper Outcomes

The scientific production related to DL and COVID-19 in 2020 (316 papers) has been co-authored by 1,654 researchers. The median co-authors per paper was four (range 1 - 39 co-authors). Moreover, we detect that some researchers co-author up to four papers.

**FIGURE 2.**
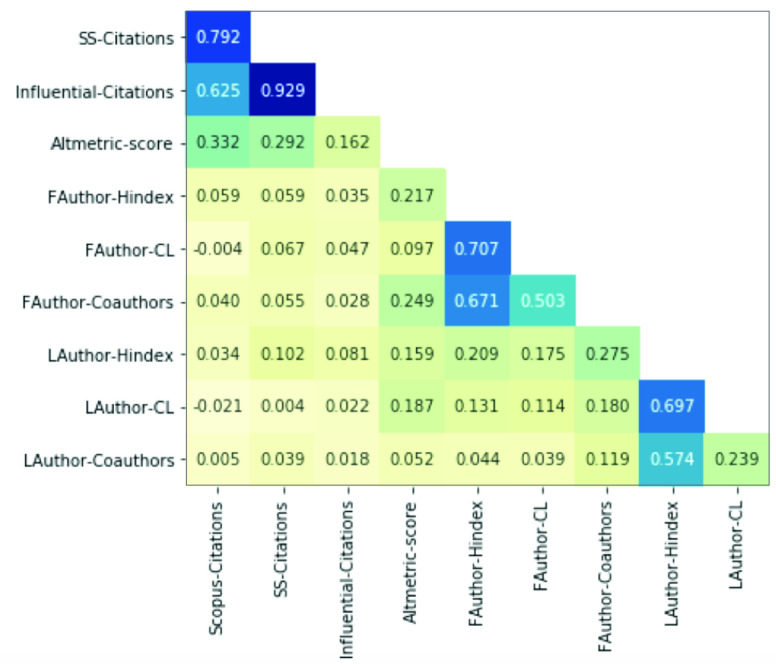
Correlation matrix between papers outcome and authors attributes. Correlation results from comparing authors’ information (h-index, number of co-authors, and the career length) and paper outcome metrics (citation count, online attention, and influential citations).

We evaluate the potential correlation between the author’s attributes and the citation count or the attention one paper has received (Fig. 2). Regarding the author’s information of each article, we analyze the following attributes for the first (*FAuthor*) and last (*LAuthor*) author: h-index (*Hindex*), number of co-authors (*Coauthors*), and the career length, i.e., the number of years since the first publication of a given author (*CL*). As for the papers, we consider citation count in Scopus (*Scopus-citations*) and Semantic Scholar (*SS-citations*), a record of online attention (*Altmetric-score*), as well as the Semantic Scholar’s metric of highly influential citations (*Influential-Citations*).

We find correlations between some of the mentioned attributes (Fig. 2). From these coefficients, it is worth highlighting the followings aspects:

• There is a strong positive correlation between the influential citations and the citation count registered in Semantic Scholar ( ${r}$(314) = 0.93, ${p}$ < 0.0001). This correlation is expected as both metrics are obtained from the same source.
• There is a strong positive correlation between the citation count in Scopus and the citation count registered in Semantic Scholar ( ${r}$(314) = 0.79, ${p}$ < 0.0001). As mentioned before, the number of citations registered in Semantic Scholar is higher than those registered in the Scopus database for the same document. Nonetheless, this metric is related in both databases suggesting that the tendencies are maintained.
• The author’s h-index shows a strong positive correlation to the career length (first and last authors: ${r}$(314) = 0.70, ${p}$ < 0.0001), and the number of co-authors (first author: ${r}$(314) = 0.67, ${p}$ < 0.0001; last author: ${r}$(314) = 0.57, ${p}$ < 0.0001). This correlation is also expected as a scientist with a longer career would have more publications with different coauthors. This trajectory and a high citation impact in most publications would result in a high h-index.
• There is a weak correlation between the citation count and the attention a paper received in social networks (*Altmetric-score*) (Scopus ${r}$(314) = 0.33, ${p}$ < 0.0001; Semantic Scholar ${r}$(314) = 0.30, ${p}$ < 0.0001). Thus, even though social media can be a platform for sharing research, our results show that the attention received by these platforms has only a 30% impact on the number of citations a document receives.
• There are no correlations between the author’s attributes and the citation count a paper received ( ${p}$ > 0.05). Hence, there is no direct relation between considered author attributes and the impact one paper achieves.

##### b: Author’s Affiliation Country

**FIGURE 3.**
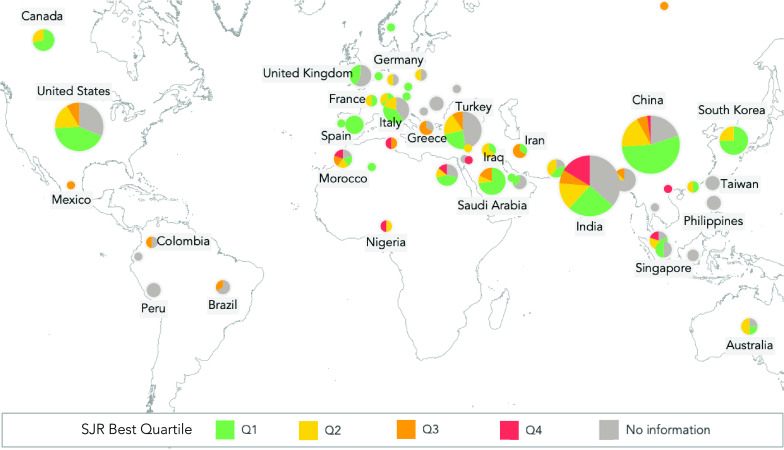
First author’s country of affiliation and quartile ranking of the 316 papers published regarding COVID-19 and DL. The number of affiliated authors in each country (pie chart) and the quartile ranking of each paper (colors).

We consider the country of affiliation of the first author for each paper to determine which countries contribute to our research topics (Fig. 3). Our results show that authors have worked primarily from five countries: India (55), China (50), the USA (34), Turkey (21), and South Korea (12). From these countries, China (54%) and South Korea (75%) stand out as evidence of the most significant proportion of published documents with the highest impact on quartile ranking.

**FIGURE 4.**
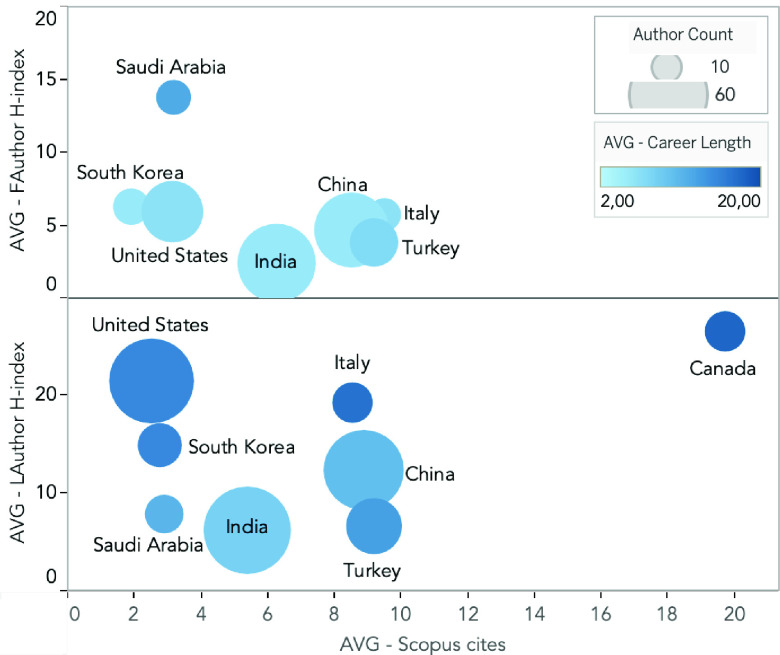
Comparison of first and last authors’ affiliation country, personal, and paper metrics from the 316 papers regarding COVID-19 and DL. The figure depicts the number of authors affiliated per country (size of pie chart), the average author’s career length (color scale), the average Scopus cites (x-axis), and the average author h-index (y-axis).

Finally, we explore the relation between the author’s affiliation country, career length, h-index, and citation counts registered in the Scopus database (Fig. 4). Our results show that the last authors from Canada (11) present an average h-index of 26.45, average citations of 19.73, and average career length of 18 years. Based on this information, these authors seem to have published the most relevant research related to DL and COVID-19. Likewise, the last authors from the USA (48) showed an average h-index of 21.40, average citations of 2.52, and an average career length of 15 years. Even though authors working in the USA had fewer citations per paper, this could be influenced by the number of authors registered under this affiliation. When analyzing the same information from the first authors, researchers from Saudi Arabia (11) had an average h-index of 13.73, average citations of 3.18, average career length of 12 years. These authors have a comparable h-index, citation count, and career length to researchers from Canada and the USA.

#### 2) Impact

##### a: Number of Citations

We find a statistically significant difference between the number of days a document has been available to the public considering the *Creation Date* (publication date in the source reported by *Crossref* database; mean 168.67, SD 66.37) and the indexation date (date of indexation in Crossref; mean 76.30, SD 54.00), being higher in the first ( ${p}$ < 0.0001). Consequently, we use the *Creation Date* of each of the 316 documents to compare with the average cites those papers have received until February 2021 (see Table 3). As expected, only two papers were published on the scope of COVID-19 and DL at the start of the pandemic. In contrast, the number of papers increases as time evolves and generate scientific interest. Papers published at the first three months of the pandemic have received the most significant scientific attention regarding the average number of citations per paper. All of them have been cited according to the *Rate of cited papers* column. Likewise, 40 papers were published in December 2020, and they have received three references until the date we extracted the data (February 2021). Hence, there is a strong positive correlation between the amount of time a paper has been accessible to the number of citations it receives ( ${r}$(314) = 0.71, ${p}$ < 0.0001).

**TABLE 3:** Citations Metrics of the 316 Articles Published Between March and December 2020 Regarding DL and COVID-19. The Table Depicts the Number of Papers Published Each Month, the Number of Citations Registered in the Scopus Database, and the Average Citations Received by Each Cited Paper

Publication month	Papers count	Total Scopus cites	Num. of cited papers	Avg. cites per cited paper
March	2	299	2	149.5
April	4	288	4	72.0
May	17	498	17	29.3
June	20	225	17	13.2
July	39	343	34	10.1
August	30	96	24	4.0
September	42	69	25	2.8
October	53	44	16	2.8
November	69	39	12	3.3
December	40	3	2	1.5

Articles published from August till December have received fewer citations compared to work published in earlier months. For instance, November 2020 has the highest published work regarding COVID-19 and DL (Table 3). However, only 12 papers are referenced elsewhere and have received an average of 3.3 cites per work. In comparison, the 34 articles published in July have received an average of 10.1 cites per work. As mentioned above, time is correlated to the number of citations a paper receives. Nonetheless, the number of published papers available to the public can be a variable that affects the number of references an article receives. Indeed, there were relatively few papers to be cited at the beginning of the COVID-19 pandemic, so they gained high scientific attraction. This particular effect is provoked by the COVID-19 pandemic and the knowledge surrounding it, which is beyond the scope of our current work.

##### b: Amount of Readers and Twitter Mentions

**FIGURE 5.**
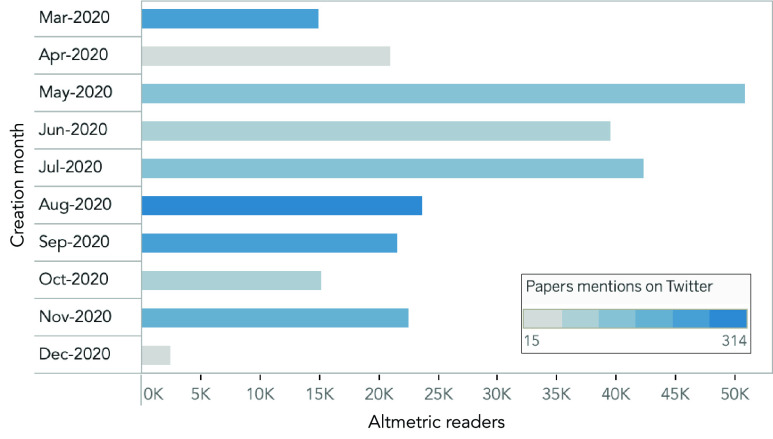
Distribution of Altmetric readers and number of Twitter mentions of the 316 papers published between March and December 2020 regarding DL and COVID-19. The number of readers as registered on Mendeley, Connotea, and Citeulike platforms (in bars). The number of times a paper is mentioned on Twitter (in color scale).

We analyze the number of Altmetric readers and Twitter mentions that each paper has, as reported by the Altmetric database (Fig. 5). The *Altmetric* API reports the total number of readers that one paper receives on *Mendeley*, *Connotea* and *Citeulike* platforms [42]. Articles created in May, July, and June 2020 present the highest number of readers, with over 35 thousand Altmetric readers. On the other hand, papers created in March, August, and September 2020 show the highest number of Twitter mentions, with over 300 mentions. Hence, the number of readers and Twitter mentions are not proportional to the time elapsed since the article’s creation date. This situation is typical for Twitter mentions since the attention on social media decreases with time. Moreover, there is no direct relation between both metrics, meaning that one paper is susceptible to having few reads with great social media attention and vice versa, as seen for the papers published in March and June 2020.

#### 3) Paper’s Features

##### a: Document Type

**FIGURE 6.**
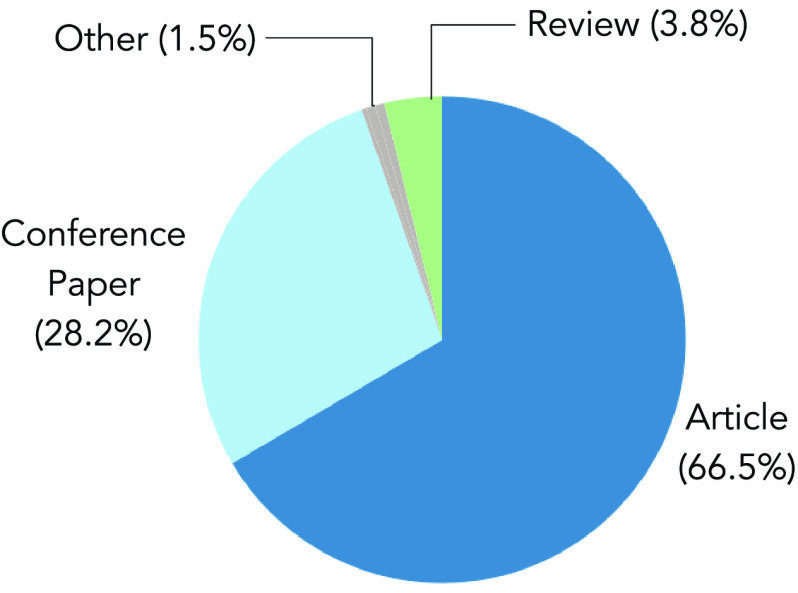
Document Type classification for the 316 analyzed papers related to COVID-19 and DL. Percent of papers categorized as articles, conference papers, reviews, and other types of documents.

Among the 316 analyzed documents, 66.5% are articles, 28.2% are conference papers, 3.8% are reviews, and the remaining 1.5% are other types of documents (Fig. 6). These results demonstrate the author’s need to publish their research immediately instead of waiting for a future conference presentation [48]. However, in the present studied areas of DL and COVID-19, the prevalence of articles, conference papers, and reviews over other types of documents is kept regarding the macro-disciplinary areas [49].

##### b: Paper’s Accessibility

**FIGURE 7.**
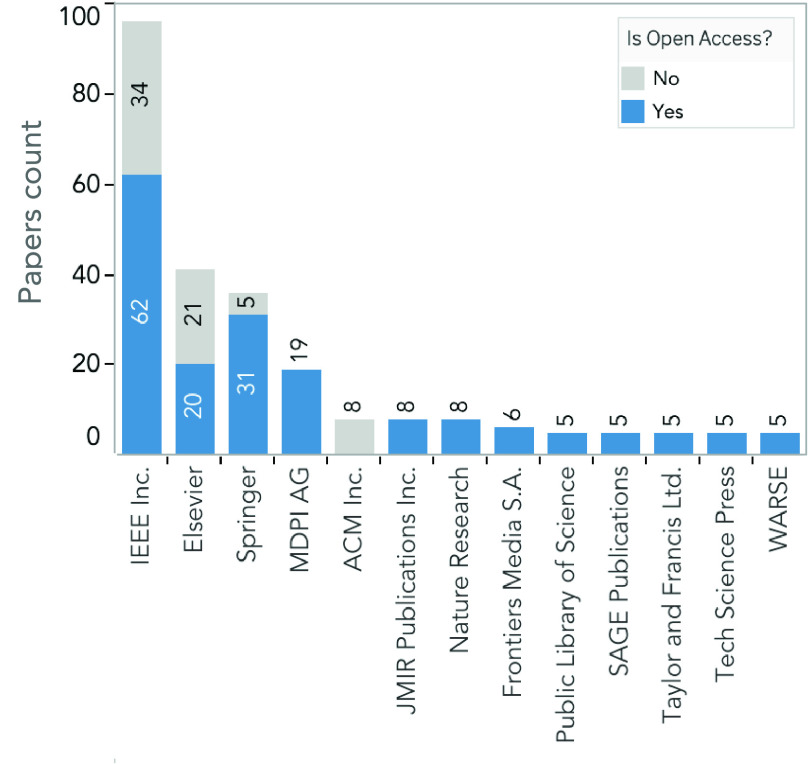
Information of the editorials that published the 316 papers regarding COVID-19 and DL between March and December 2020. The number of papers per editorial group (in bars), and if the papers are Open Access (blue) or not (gray).

Documents can be accessed either freely (i.e., Open Access) or based on subscription. We expect that work related to the COVID-19 to be of significant interest and should be available for the research community as soon as possible to save time in the fight against SARS-CoV-2. Therefore, one may think that Open Access articles would be the preferred approach to publish work in our field of study. We analyze the editorials that published the 316 documents considered in this study (Fig. 7). Our results evidence that most of the papers were Open Access. However, there are apparent exceptions where papers published in some editorials declined this free accessibility. Such is the case of the editorials Elsevier, and the Association for Computing Machinery (ACM) Incorporated, in which over 50% of the published work decline Open Access. This difference could be partly because of the economical price authors have to pay to the editorials to make their research Open Access.

##### c: Paper’s Keywords

**FIGURE 8.**
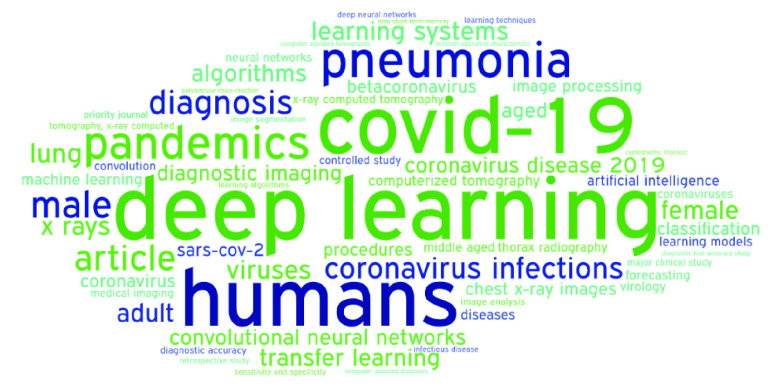
Main keywords from the 316 papers regarding COVID-19 and DL published between March and December 2020.

We further analyze the most representative keywords used by authors through a word cloud chart (Fig. 8). We filter keywords with higher or equal than fifteen occurrences to fine-tune our goal. As expected, the most common keywords are *deep learning, COVID-19, humans, pandemics*, and *pneumonia*. The latter is the most frequent disease derived from the COVID-19.

#### 4) Publishing Information

##### a: Publishers

When analyzing where the work was published, we find that four editorials enclose more than 50% of the papers regarding COVID-19 and DL (Fig. 7). In this regard, the editorial Institute of Electrical and Electronics Engineers (IEEE) concentrates most of the documents with over one hundred papers (~30%), followed by Elsevier (41 articles, ~13%), Springer (36, ~11%), and MDPI (19, ~6%). The rest of the papers are almost uniformly distributed among other editorials.

##### b: Publishing Sources

In addition to the publishers, we analyze the sources where papers have been published. The results show that the selected 316 papers have been published in 184 different publishing sources. The sources with the higher number of papers in the context of our study are Chaos, Solitons and Fractals, IEEE Access, and Applied Intelligence (Table 4).

**TABLE 4:** Top-10 of the Most Popular Sources Where the 316 Papers Were Published Between March and December 2020. The Table Describes the Type of Source, and the Number of Papers Published in Each Source

Source	Type	Count
Chaos, Solitons and Fractals	Journal	16
IEEE Access	Journal	12
Applied Intelligence	Journal	8
European Radiology	Journal	7
IEEE Journal of Biomedical and Health Informatics	Journal	6
Computers in Biology and Medicine	Journal	5
PLoS ONE	Journal	5
International Journal of Advanced Trends in Computer Science and Engineering	Journal	5
Applied Sciences (Switzerland)	Journal	5
4th International Symposium on Multidisciplinary Studies and Innovative Technologies, ISMSIT 2020 - Proceedings	Conference and Proceedings	5
Scientific Reports	Journal	5

##### c: Citations Per Publisher

**FIGURE 9.**
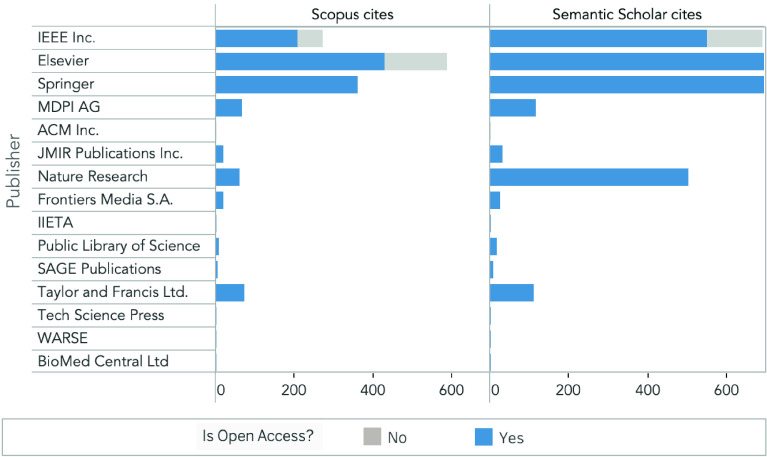
Comparison between the number of citations of the 316 papers registered in Scopus and Semantic Scholar. Color bars define whether the papers are Open Access (blue) or not (gray).

Furthermore, we compare the number of citations registered in two bibliographic databases (Fig. 9). We find a statistically significant difference when comparing the number of citations per document registered in Scopus (mean 6.03, SD 18.69) to Semantic Scholar (mean 12.77, SD 43.41), being higher in the latter ( ${p}$ = 0.013). This difference can be seen when IEEE, Elsevier, and Springer register over 600 citations at the Semantic Scholar database, while these editorials register less than 600 citations at the Scopus database (Fig. 9). Additionally, even though IEEE published the highest amount of papers (Fig. 7), these documents did not have the same influence over the research community as work published by Elsevier when accounting for the number of citations each paper has received. This marked difference could be explained by the strategies applied by each editorial for the broadcasting of their information. Further work is needed to understand this dynamic.

##### d: Publishing Sources Country and Quartile

**FIGURE 10.**
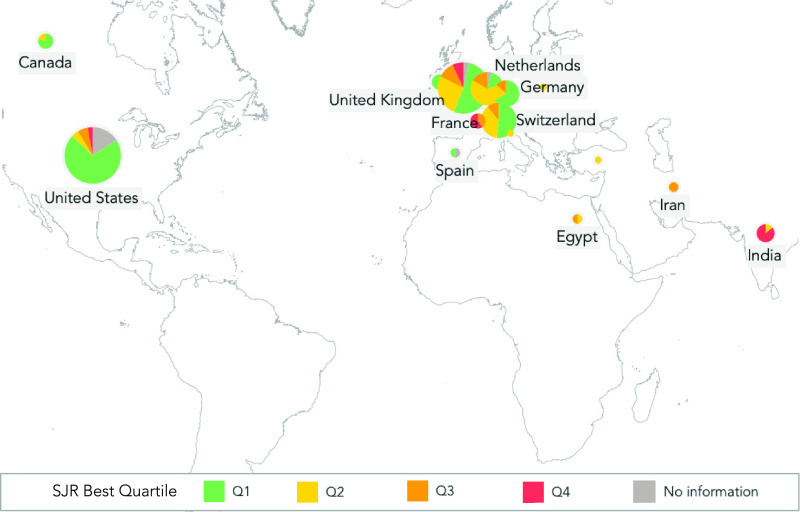
Geographical distribution and Quartile ranking of the publishing sources of the 316 papers regarding COVID-19 and DL. The number of publishing sources which headquarters are registered in each country (pie chart), and the quartile ranking each source has (colors).

Using information provided by the Scimago database, we analyze the publishing source’s headquarters country as well as its quartile ranking (Fig. 10). Our results show that five countries stand out by grouping most of the publishing sources: United States (69), United Kingdom (57), Switzerland (27), Netherlands (23), and Germany (15). Furthermore, the USA also concentrates most of the publishing sources ranked in the Q1 quartile.

### B. Qualitative Analysis

In this subsection, we present the results about qualitative analysis grouped in five main categories: (1) *High-scored papers*, (2) *Impact*, (3) *Multidisciplinarity*, (4) *Technical information*, and (5) *Performance*.

#### 1) High-Scored Papers

**FIGURE 11.**
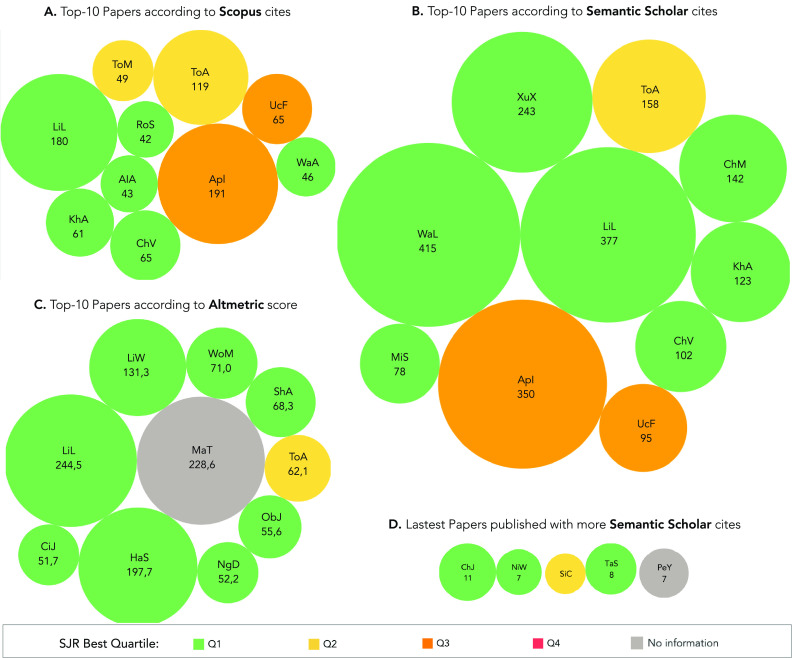
Top-27 papers. Papers are selected based on their citation scores in: a) the Scopus and b) Semantic Scholar databases; as well as c) their influence in social media given by the Altmetric score. Finally, d) we select the papers with high number of citations and that were published in the last three months. Abbreviations: AlA [33]; ApI [29]; ChJ [31]; ChM [25]; ChV [27]; CiJ [30]; HaS [56]; KhA [26]; LiL [37]; LiW [57]; MaT [51]; MiS [52]; NgD [58]; NiW [55]; ObJ [59]; PeY [35]; RoS [34]; ShA [60]; SiC [32]; TaS [36]; ToA [50]; ToM [61]; UcF [62]; WaA [53]; WaL [54]; WoM [63]; XuX [64].

We select the most outstanding papers (top-10) from Scopus, Semantic Scholar, and Altmetric databases (Fig. 11). Our results show some papers [37], [50] that figured within the top-10 in the three databases. These papers [37], [50] have several citations and significant online attention (Altmetric score). However, these papers have different citation counts between the Scopus and the Semantic Scholar databases (Fig. 11 A and B). As previously mentioned, Semantic Scholar registers a higher number of citations per paper compared to Scopus. For instance, Ref. [37] registered 180 citations at the Scopus database and 377 at Semantic Scholar. Furthermore, some papers reach the top-10 place of only one database [34], [51]–[54]. Of particular interest, Ref. [54] has the first place in the Semantic Scholar database with 415 citations. On the other hand, this work only registered 15 citations on the Scopus database, giving it the 24th place within our list of papers for that database.

We analyze separately the papers published in the last three months, as the number of citations is dependent on the time a document is available to the public (Fig. 11 D). Our results show that there were five documents with a representative number of citations considering the short time of availability [31], [32], [35], [36], [55]. These documents have at least five citations registered at the Semantic Scholar database. Within these, the work presented by Chen *et al.* registers the highest number of citations (12 for Scopus and 11 for Semantic Scholar) for such a short time [31].

#### 2) Impact

##### a: Contribution Area and Scope

The top-27 selected papers are cataloged in six contribution areas (Table 5). The most popular category is *Diagnostics* (19/27), with authors aiming to find an alternative towards detecting COVID-19 patients using DL and imaging (CT-scans, X-rays or lung ultrasound (LUS) images). The remaining five categories present one or two papers each, showing that the scientific community improved diagnosis methods. When considering the scope of the top-27 studies, most of the work is done with global information (18/27), followed by publications centering their research on data from China (5/27). Research that focused on diagnosis and used global information represents 52% of the top-27 papers. These papers obtained images from free datasets and highlighted the need for more images from COVID-19 patients to reduce the observed error. In contrast, particular researchers have focused on specific regions, especially in the diagnosis, prognosis, and forecasting categories [27], [31], [55].

**TABLE 5:** Qualitative Analysis of Top-27 Papers. Detailed Information Regarding the Work That is Being Analyzed (Ref.), Scope of the Study (Region), Contribution Area, Prediction Task, the Architecture of the Model, if the Code is Available Online, Influence on Public Policies, Communication Media (CM) or Both, if the Context of the Citation is a Mention (M), Support (S), or Contrasting (C)

Ref.	Scope	Contribution Area	Prediction task	DL method: model	Online code	Influence	Citation Context
[25]	Global	Diagnostics	Image Classification	CNN: MobileNet v2, SqueezeNet, ResNet101, ResNet18, DenseNet201 CheXNet, Inceptionv3 and VGG19		N/A	S-3, M-119
[26]	Global	Diagnostics	Image Classification	CNN: Xception	Yes	No	M-145, C-1
[27]	Canada	Forecasting	Regression of COVID cases	RNN: LSTM		No	M-78
[29]	Global	Diagnostics	Image Classification	CNN: VGG19, MobileNet v2, Inception, Xception and Inception ResNet v2		No	S-3, M-385, C-1
[30]	Global	Diagnostics	Image Classification	CNN: VGG16		CM	M-14
[31]	China	Diagnostics	Image Classification	CNN: Resnet50 + UNet++	Yes	CM	M-121
[32]	Global	Diagnostics	Image Classification	CNN: VGG16	Yes	N/A	M-1
[33]	Global	Diagnostics	Image Classification	CNN: EfficientNet-B0		N/A	M-21
[34]	Italy	Diagnostics	Image Classification	CNN: Regularised Spatial Transformer Networks	Yes	CM	M-138
[35]	Global	Diagnostics	Image Classification	CNN: DenseNet121	Yes	No	M-1
[36]	Global	Diagnostics	Image Classification	CNN: Resnet50		No	M-6
[37]	China	Diagnostics	Image Classification	CNN: Resnet50	Yes	Both	M-301
[50]	Global	Therapeutics	Prediction of molecules score	Not details		Both	M-109
[51]	Global	Misinformation	Posts classification	RNN: LSTM		CM	M-1
[52]	Global	Diagnostics	Image Classification	CNN: COVNet based on ResNet18, ResNet50, SqueezeNet, and DenseNet161	Yes	N/A	S-1, M-284, C-1
[53]	Global	Diagnostics	Image Classification	CNN: VGG16		N/A	M-59
[54]	Global	Diagnostics	Image Classification	CNN:COVID-Net	Yes	CM	S-1, M-289
[55]	China	Prognostic	Prediction of morbidity and mortality outcomes	CNN: Inception Net V3 + ChexNet	Yes	No	S-1, M-2
[56]	Global	Diagnostics	Image Classification	CNN: DenseNet121		CM	M-27
[57]	China	Prognostic	Risk stratification classification	FNN: Deep Learning Survival Cox	Yes	CM	S-2, M-54
[58]	Global	Therapeutics	Prediction of 3D molecules properties	CNN: MathDL model		CM	S-1, M-1
[59]	USA	Diagnostics	Risk stratification classification	CNN: Word2vec		CM	M-6
[60]	Global	Misinformation	Ethical behavior classification	FNN: MLP	Yes	CM	M-1
[61]	Global	Diagnostics	Image Classification	CNN: MobileNet v2	Yes	No	M-83, C-1
[62]	Global	Diagnostics	Image Classification	CNN: Bayes-SqueezeNet		No	M-117
[63]	N/A	Imaging^*^	N/A	N/A		CM	M-3
[64]	China	Diagnostics	Image Classification	CNN: ResNet18		No	S-1, M-206

^*^ : Review. N/A: not applicable. For the Influence column, N/A relates to papers that were not found in Altmetric website

##### b: Medical Application or Usability

From the top-27 papers, six papers can be applied to a medical discipline. For instance, *Diagnosis* using a DL model and CT-scan images achieved comparable performance with expert radiologists in a shorter time [31]. Implementing this model could improve the diagnosis of COVID-19 in hospitals with a lack of personnel. Likewise, AI for the electronic triage process can be used as a diagnostic tool to prioritize patients with a higher probability of screening positive for COVID-19 based on self-reported symptoms [59].

DL models that predict the prognosis of COVID-19 patients based on clinical characteristics at admission [57] or CT-scan images and clinical features [55] could help catalog negative, mild and severe cases. Implementing these models could help detect patients at risk of severe illness and ensure proper care as early as possible. Finally, to set the base for future drug discovery towards inhibition of SARS-CoV-2, DL models were used to rank the binding affinities of inhibition structures to the SARS-CoV-2 main protease [50], [58].

##### c: Influence on Public Policies or Mainstream Media

We analyze the type of influence the top-27 papers have had over public policies or mainstream media based on information found in the Altmetric database (Table 5). Altmetric tracks a global range of sources looking for references to published research. From the top-27 documents, 22 papers were found in the Altmetric database when we collected our data (February 2021). Our results show that at least 59% (13/22) of these papers influenced public policies or mainstream media. From this list, the work presented by Li *et al.* stands out as it has been referenced by 12 important press articles and one WHO public policy document [37].

##### d: Citation Context Analysis

As previously mentioned in subsection IV-C2, the citation count metric on its own could lead to wrong conclusions regarding the impact a paper has generated. Hence, we explore the citation context based on the categorization provided by the *scite.ai* website (Table 5). This metric accounts for all references one paper receives within another research document. Thus, if one paper is cited four times in different parts of the same document, four references are counted because each may have a different context. As a result, this metric is usually not aligned with the one reported by WoS or Scopus databases. Our results show that all top-27 papers received at least one citation under the category of *Mentioning* (Table 5). However, without loss of generality, we can say that the *Supporting* citation type is the most relevant because it indicates that one paper is used to leverage other studies and findings. Such is the case of 30% (8/27) of the papers on the selected list.

#### 3) Multidisciplinarity

**FIGURE 12.**
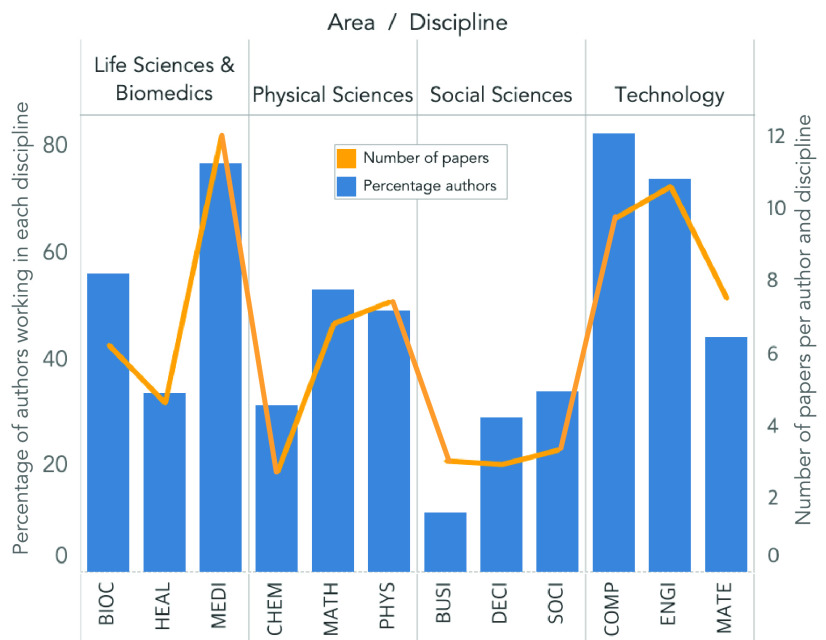
Research areas and number of publications per area from the authors of the top-27 papers. The percentage of authors working in each area (blue) and the number of publications per paper and area (orange line).

We analyze the composition of the research teams from the top-27 papers. For this purpose, we retrieve the authors’ list of publications and explore their research areas using the Scopus Author ID tool. We group the publications in four research areas (*Life Sciences & Biomedics*, *Technology*, *Physical Sciences*, and *Social Sciences*) and three fields within each area. We compute a percentage of authors that

contribute to the publication of an article in each field. We further determined the number of published papers per author and area. The results disclose that most of the authors work in computer sciences (83%), followed by medicine (77%) and engineering (74%) (Fig. 12). Furthermore, on average, most of the published papers are related to medicine (12), engineering (11), and computer sciences (10) (Fig. 12). These results give insights into the multidisciplinarity of authors participating in DL and COVID-19 research.

#### 4) Technical Information

##### a: Prediction Task

We identify the main prediction task from each top-27 paper by reading and analyzing each one individually (Table 5). The most common task is classification (22/27), followed by forecasting (4/27), and none task (1/27) [63].

Regarding the group of paper addressing Classification task, Image Classification is the preferred DL prediction task carried out by authors (82%). Indeed, authors demonstrate that DL methods had been useful for detection COVID-19 in patients by using imaging (CT-scans or X-rays) [25], [26], [29]–[37], [52]–[54], [56], [61], [62], [64]. To complete the group of papers that addressed the Classification task, authors have tried to detect fake COVID-19 information using textual and binary classification [51]. Also, a DL model is used to predict whether the study’s respondents perceived unethical behaviours as justifiable, important aspect to be considered in times of crisis [60]. Likewise, authors propose a model for risk stratification classification [57], [59].

The four remaining papers belong to the group addressing Forecasting task. These papers have estimated COVID cases [27], predicted docking scores for chemical structures into an active site of novel SARS-CoV-2 Mpro [50], predicted COVID-19 morbidity and mortality outcomes enabling the early diagnosis of patients with COVID-19 pneumonia [55], and predicted 3D molecules properties [58].

##### b: DL Method and Model

CNN is the most popular DL method (21/27) used in the top-27 papers (Table 5). CNN is generally used for image processing applications, and its popularity embraces our previous findings of authors aiming to improve diagnostic methods towards COVID-19. Furthermore, RNN is also used by authors (2/27). RNN is designed to recognize sequences and patterns such as speech, handwriting, and text. A particular RNN method is Long Short-Term Memory (LSTM), which can be used for both sequences and pattern recognition and image processing applications [65]. By each method, there are some pre-trained architectures to do one or more ML tasks. Pre-trained DL models are trained networks that use large datasets and avoid over-fitting in most cases [32].

##### c: Online Code

As observed in Table 5, the authors of twelve papers [26], [31], [32], [34], [35], [37], [52], [54], [55], [57], [60], [61] have published the software code of their developments and/or experiments. Code, data, and model availability is important to ensure the reproducibility as well as to improve the quality and performance of the proposed models.

#### 5) Performance

From the top-27 papers, we analyze the performance and architecture of 24 papers (Table 6). Three papers are excluded from these analyses due to a lack of detailed architecture and performance indicators [50], [58] or a deficit of DL implementation [63]. Furthermore, some proposals involved the evaluation of more than one architecture. Hence, Table 6 shows the architecture that presented the best performance as reported by the authors. From the 24 papers, we highlight the following relevant characteristics:

a) *Architecture:* The DL models that show the best performance are: VGG16 [30], [32], [53], ResNet50 [31], [36], [37], Densenet121 [35], [56] and MobileNet v2 [29], [61]. Furthermore, we observe that, in some architectures, authors added mechanisms such as:
  • Location-attention to improve the image discrimination of specific organ structures between healthy and compromised patients [32], [64].
  • Synthetic augmentation method to counter the scarcity or lack of datasets [53].
  • Pre-processing datasets using techniques of image processing [33] or segmentation [31], [34], [56], [64]. These techniques can help the classification algorithms focus on the target by excluding external factors [56]. Other pre-processing methods we find are wavelet transformation [27], equalization [30], Fuzzy technique, and Stacking technique [61].
  • Improving datasets by image augmentation tasks [25], [54], [62]. Comparing data without image augmentation vs. image augmentation, the latter achieved the best performance [25].
b) *Data type:* “images” is the most popular data type within the 24 papers. In particular, chest X-Ray (CXR) images have been used by more than 50% of the researchers, followed by computed tomography (CT) with 21% and LUS images in one research [34]. Furthermore, “time series” is used to predict the evolution of the COVID-19 clinical features [27]. In combination with images, “time series” is applied to make early decisions by improving the ability to identify patients at higher risk of complications [57]. Data types, including Instagram and Twitter posts [51], patients’ clinical notes [59], COVID- 19 articles [35], and survey responses [60], have also been used by researchers of the 23 papers.
c) *Data size:* the 24 papers can be grouped into three categories based on their data size: less than 1000 [30], [36], [51], [60], [61], [64], between 1000 and 5000 [25], [26], [29], [32], [33], [37], [52], [53], [56], [57], [62], and more than 5000 [31], [34], [35], [54], [55], [59]. Only one paper did not specify the data size used for their analyses [27].
d) *Class number:* more than 92% (22/24) of the research has focused on the classification task. Half of these papers have obtained the best performance with two classes [25], [26], [29], [31], [35], [36], [51]–[53], [56], [60], obtaining the best performance when predicting if an observation is COVID-19 or any other condition. From the remaining papers, nine used classification of three classes, being the most common COVID-19, pneumonia, and healthy. [30], [33], [37], [54], [55], [57], [61], [62], [64] Lastly, a study obtained the best performance with four classes [34] and other with five classes [32].
e) *Evaluation metrics:* all 24 papers have used at least one of the traditional evaluation metrics such as accuracy, specificity, positive predictive value, and sensitivity. Most papers (18/24) have analyzed further evaluation metrics as F1 score [25], [26], [30], [33]–[36], [51], [59], [61], [62], negative predictive value (NPV) [31], [55], [56], or area under the receiver-operating characteristic curve (AUC) [37], [57], [59], [60]. The only work analyzing a different evaluation metric considered a Root Mean Square Error (RMSE) [27].

**TABLE 6:** Analysis of the Performance of 24 of the Top-27 Papers. Detailed Information Regarding the Work That is Being Analysed (Ref.); Architecture Which Reports the Best Performance (Architecture); Data Type That Was Analyzed; Data Size in Thousands (k); Number of Classes for Classification (Class No.); Accuracy (Acc.); Specificity (Spe.); Positive Predictive Value (PPV); Sensitivity (Sen.); and Other Metrics

Ref.	Architecture	Data type	Data size (k)	Class No.	Acc.	Spe.	PPV	Sen.	Other metrics
[25]	DenseNet201	CXR images	~3.48	2	~99.7	~99.55	~99.7	~99.7	F1: ~99.7
[26]	CoroNet based on Xception	CXR images	~1.25	2	~98.3	~98.60	~98.3	~99.3	F1: ~98.5
[27]	LSTM model-1	Time series	NE	N/A	~92.7				RMSE: ~45.7
[29]	MobileNet v2	CXR images	~1.44	2	~96.8	~96.46		~98.7	
[30]	VGG16	CXR images	~0,39	3	~86.0	~93.00	~86.0	~86.0	F1: ~86.0
[31]	ResNet50 + Unet++	CT images	~30.76	2	~96.0	~94.00	~94.2	~98.0	NPV: ~97.9
[32]	VGG16 + Location-attention	CXR images	~2.14	5	~87.0				
[33]	EfficientNet-B0 + 2D curvelet transform + CSSA	CXR images	~2.90	3	~99.7	~99.81	~99.6	~99.4	F1: ~99.5
[34]	Reg-STN + SORD	LUS images	~58.92	4			~70.0	~60.0	F1: ~65.1
[35]	DenseNet121	COVID-19 Articles	~5.38	2	~99.6	~99.90		~99.8	F1: ~99.7
[36]	COVID-SDNet based on Resnet50^(*)^	CXR images	~0.85	2	~81.0	~85.00	~84.2	~76.8	F1: ~80.1
[37]	COVNet based on Resnet50^(*)^	CT images	~4.35	3		~96.00		~90.0	AUC: ~96.0
[51]	Covid Model based on LSTM	Instagram posts	~0.60	2			~78.0	~66.4	F1: ~71.7
[52]	COVNet based on SqueezeNet	CXR images	~5.00	2		~92.90		~98.0	
[53]	CovidGAN based on VGG16 + GAN	CXR images	~1.12	2	~95.0	~97.00	~96.0	~90.0	
[54]	COVID-Net^(*)^	CXR images	~13.98	3	~93.3		~98.9	~91.0	
[55]	HUST-19 based on Inception Net v3	CT images	~19.68	3		~99.80	~99.5	~85.0	NPV: ~93.9 AUC: ~97.8
[56]	Densenet121 Hybrid 3D	CT image	~2.72	2	~89.5	~95.10	~85.3	~75.1	NPV: ~90.9 AUC: ~93.8
[57]	Deep Learning Survival Cox model	Clinical features	~1.59	3		~95.00		~95.0	AUC: ~91.1
[59]	Word2vec	Patients’ Clinical notes	~6.81	N/A			~75.4	~45.3	AUC: ~73.2 F1: ~56.6
[60]	MLP	Survey’s responses	~0.20	2	~90.0	~50.10	~76.5	~86.3	AUC: ~79.6
[61]	MobileNet v2 + SqueezeNet + SMO^(*)^	CXR images	~0.46	3	~99.3		~99.7	~99.3	F1: ~99.5
[62]	Bayes-SqueezeNet	CXR images	~4.61	3	~98.3	~99.13			F1: ~98.3
[64]	Resnet18 + Location-attention	CT images	~0.62	3	~86.7				

N/A: not applicable.

^(*)^ : Performance for COVID- 19 class

## VI. Conclusion

Our work has addressed a bibliometric analysis of scholarly production published during 2020 that has applied DL to combat the COVID-19 pandemic. This bibliometric analysis has focused on quantitative and qualitative indicators that give insights into the distribution, organization, impact, relevance, limitations, and contributions of the scientific literature produced around DL and COVID-19. Thus, our work can help the scientific community keep abreast of the research and extend or apply their findings in the ongoing battle against COVID-19.

The quantitative analysis described the main characteristics of the articles and their related entities, including authors, countries, institutions, and journals. Our study has analyzed the scholarly production indexed by Scopus in 2020, according to elemental data like monthly evolution, document type, publishing sources, and keywords. Furthermore, from the selected list of papers (316) regarding COVID-19 and DL, we highlighted the relationship between several characteristics like papers and authors, author’s country and quartile ranking, authors’ affiliation country and paper metrics, social and citations metrics of the articles, as well as geographical distribution and quartile ranking of the publishing sources. Likewise, we used different databases (Scopus, Semantic Scholar, Altmetric, and Crossref) to do a multidimensional analysis. Here, we found connections among selected literature that helps to understand the publication characteristics and contributions.

We selected articles with the highest impact considering the number of cites or social mentions (referred to as top-27 papers) to carry out the qualitative analysis. We deepened the analysis to highlight technical information, research outcomes, contribution in applying DL techniques, and alternative indicators of the impact of scientific publications in metrics based on citations and digital footprint at the document and author levels. The wealth of information generated on the web around one research document can give us valuable insights into the research impact and influence on society, governments, academia, public and private organizations. Specifically, our analysis revealed that the literature addresses different problems and concerns about COVID-19 and outlines approaches to mitigate the adverse effects of the pandemic by using DL methods and, in some cases, by reusing pre-trained models. This research contributes to increasing the knowledge about the behavior, diagnostic, treatment, propagation, and variation of this virus. From the list of contribution areas of DL techniques, diagnostics stands out as being globally applicable. However, other studies have focused on specific regions, especially diagnosis, prognosis, and forecasting categories. In any case, experiments and performance metrics demonstrate the potential use of DL methods across different domains. In synthesis, DL algorithms were fruitful to recognize patterns in data, whether there is a scarcity or abundance of them, to support and help human experts to make better decisions.
